# Seropositivity of Anti-*Toxoplasma gondii* and Anti-*Neospora caninum* Antibodies in Cattle Intended for Human Consumption in an Amazonian Area of North Brazil

**DOI:** 10.3390/tropicalmed8070359

**Published:** 2023-07-10

**Authors:** Victor Hugo Alves Sousa Formiga, Felipe Boniedj Ventura Alvares, Mariana Moreira Anjos, Jefferson Vieira Freitas, Daiane Peixer Silva, Roberta Nunes Parentoni, Arthur Willian Lima Brasil, Gláucia Diojânia Azevêdo Medeiros, Thais Ferreira Feitosa, Vinícius Longo Ribeiro Vilela

**Affiliations:** 1 Department of Veterinary Medicine, Instituto Federal da Paraíba—IFPB, Sousa 58807-630, Paraíba, Brazil; 2 Department of Veterinary Medicine, Universidade Federal de Rondônia—UNIR, Rolim de Moura 76940-000, Rondônia, Brazil; 3 Department of Veterinary Medicine, Universidade Federal da Paraíba, João Pessoa 58059-900, Paraíba, Brazil

**Keywords:** Amazon forest, cattle farming, neosporosis, toxoplasmosis

## Abstract

*Toxoplasma gondii* and *Neospora caninum* are obligate intracellular intestinal coccidia distributed worldwide, and are causative agents of toxoplasmosis and neosporosis, respectively. The aim of this study was to evaluate the prevalence of anti-*T. gondii* and anti-*N. caninum* antibodies and the factors associated with infections in beef cattle intended for human consumption in an Amazonian area of North Brazil. We collected blood samples of 387 cattle from 50 herds located in different municipalities of the State of Rondônia. An epidemiological questionnaire was distributed to farmers, with regard to nutritional, sanitary and reproductive herd management. The samples were identified, refrigerated and sent for serological analyses via IFAT (Immunofluorescent Antibody Test). Among the 387 analyzed animals, 91 (23.5%; CI 95%: 18.8–27.2) were positive for anti-*T. gondii* antibodies, with titers varying from 1:64 (75.8%) to 1:512 (2.2%). For anti-*N. caninum* antibodies, only four animals (1%; CI 95%: 0–2.7) were positive, with titers ranging from 1:400 (50%) to 1:1600 (25%). We observed a significant rate of anti-*T. gondii* antibodies in the variables “pure breed” and “contact with free-range chickens” (*p* < 0.2). There were no risk factors associated with the presence of anti-*T. gondii* or anti-*N. caninum* antibodies. In conclusion, there was a high prevalence of anti-*T. gondii* antibodies in beef cattle intended for human consumption in the State of Rondônia, Brazil, and a low prevalence of anti-*N. caninum* antibodies. Longitudinal studies can better elucidate the cause of these prevalence levels and how they could be better prevented and controlled.

## 1. Introduction

Cattle farming constitutes one of the primary economic activities in Brazil, playing a significant role in its economy. As reported by ABIEC [1], the beef cattle industry in Brazil made a substantial contribution to the country’s gross domestic product (GDP) in 2020, accounting for 10% of the total. This was achieved through the slaughter of 41.5 million heads, yielding an impressive 10.32 million tons of meat, valued at approximately USD 150.37 billion. Consequently, Brazil has secured its position as the second-largest beef producer globally. Notably, the North Region of Brazil, as documented by IBGE [2], has experienced a noteworthy surge in its cattle population, boasting a remarkable increase of 5.5%. The region’s total cattle population now stands at 52.4 million heads, with particular prominence observed in the States of Pará (22.2 million) and Rondônia (14.8 million).

In the North Region of Brazil, beef cattle ranching predominantly adopts an extensive system, whereby efforts are being made by producers to mitigate deforestation and promote sustainable livestock practices on more productive pastures within the Amazon region [3]. The productivity of Brazilian herds is influenced by multiple factors, including seasonal fluctuations in pasture availability, nutritional deficiencies, suboptimal management practices and the prevalence of parasites [4,5]. The presence of parasites, in particular, significantly hampers productive rates and reproductive performance, and leads to involuntary culling and increased mortality rates [6].

Among the parasites that infect cattle, two prominent species are *Toxoplasma gondii* and *Neospora caninum*, which are obligate intracellular coccidia responsible for causing toxoplasmosis and neosporosis, respectively. *T. gondii* primarily utilizes felids, particularly domestic cats, as definitive hosts, where it undergoes its sexual phase. During this phase, immature oocysts are excreted in the feces, and upon sporogony, they transform into infectious sporulated oocysts, which can be ingested by cattle and other intermediate hosts [7]. Although cattle exhibit natural resistance to these infections, the presence of *T. gondii* in bovine tissues highlights the significance of this infection and the potential for transmission to humans [8]. As for *N. caninum*, canids, both domestic and wild, are recognized as definitive hosts and play a crucial role in neosporosis transmission by shedding immature oocysts in their feces [9]. Nonetheless, the transplacental route is considered the primary mode of *N. caninum* transmission in cattle, leading to abortion and neonatal mortality. The endogenous route also holds importance in maintaining the parasite within cattle herds [7,10,11,12]. While neosporosis is not classified as a zoonosis, the presence of the parasite’s DNA in human umbilical cord blood, as reported by Duarte et al. [13], indicates its potential for vertical transmission and suggests the possibility of human infection.

Toxoplasmosis and neosporosis have a global distribution [10]. In Brazil, studies have revealed varying frequencies of anti-*T. gondii* antibodies in cattle, ranging from 1% to 89% [8]. However, there is a scarcity of epidemiological studies on *T. gondii* infections in cattle, specifically in the North Region of Brazil. In the State of Rondônia, the only conducted study reported a prevalence of 5.3% [14]. Regarding neosporosis, seroprevalence percentages ranging from 9.5% to 11.2% were observed in the State of Rondônia [15]. Given the significance of beef cattle ranching in the North Region, particularly in Rondônia, the zoonotic potential of bovine toxoplasmosis and the limited information available on the infection rates of *T. gondii* and *N. caninum* in cattle, the present study aimed to describe the prevalence of antibodies against these parasites and explore associated factors in cattle intended for human consumption.

## 2. Materials and Methods

### 2.1. Study Site and Sampling

We used cattle serological samples from a slaughterhouse with Federal Inspection Service in the municipality of Cacoal, State of Rondônia, between February and May of 2019. To determine the minimum sample number to be used, simple random sampling was applied, as recommended by Thrusfield [16]:

$$n=\frac{z^{2}\times P\left(1-P\right)}{d^{2}}$$

where:

*n* = number of cattle selected;

z = normal distribution value for the 95% confidence level;

P = expected prevalence of 50%;

d = 5% sampling error.

To perform adjustments for finite populations, the following formula was applied:

$$najus=\frac{N\times n}{N+n}$$

where:

*najus* = adjusted sample size;

*N* = total population size;

*n* = initial sample size.

### 2.2. Sample Population

We selected 387 cattle aged up to 24 months, from 50 different herds. The collections were conducted during 10 visits to the slaughterhouse and, in each visit, blood samples of approximately 39 animals were collected via external jugular venepuncture. Animal selection was based on systematic sampling, through which one sample was collected from every four slaughter animals. The samples were identified and stored at −20 °C until serological analyses.

### 2.3. Serological Analyses

The analyses were performed by the Laboratory of Immunology and Infectious Diseases (LIID), at the Instituto Federal da Paraíba (IFPB), Sousa campus, through immunofluorescence antibody tests (IFATs). To detect anti-*T. gondii* IgG antibodies, according to Camargo [17], tachyzoites of the ME-49 strain were used as antigens fixed in slides, with a cut-off of 1:64 [18]. To anti-*N. caninum* IgG antibodies, according to Gondim et al. [19], tachyzoites of the Nc-1 strain were used as antigens fixed on slides, with a cut-off of 1:200 [20]. The conjugate (anti-bovine IgG, labeled with fluorescein isothiocyanate, Sigma^®^, St. Louis, MO, USA) was used at a 1:700 dilution in pH 7.2 phosphate-buffered solution (PBS) containing 0.01% Evans blue. Positivity was confirmed when tachyzoites showed total peripheral fluorescence. Positive samples were submitted to two-fold sequential dilutions to determine the antibody titration.

### 2.4. Epidemiological Questionnaire 

We consulted owners’ registers at the Agência de Defesa Sanitária Agrosilvopastoril de Rondônia (IDARON) to contact participants and distribute the epidemiological questionnaire, aiming to assess of possible risk factors associated with the positivity of anti-*T. gondii* and anti-*N. caninum* antibodies. The variables and categories were the management system (intensive, semi-intensive or extensive); type of exploitation (meat, milk or mixed); type of milking (manual or mechanical); number of milkings per day (none, once a day or twice a day); presence of other animal species (cattle, horses, goats/sheep, pigs, poultry, dogs or cats); presence of wildlife (yes or no); occurrences of miscarriages during the last 12 months (yes or no); presence of rodents (yes or no); use of rodent control (yes or no); feeding on native pasture (yes or no); water source (drinking troughs or watering points); animal purchases (yes or no); pasture rental (yes or no); presence of flooded areas (yes or no); presence of maternity pens (yes or no); separation of young from adult animals (yes or no); and presence of veterinary assistance (yes or no).

### 2.5. Statistical Analyses

To assess the association between the variables from the epidemiological questionnaire and the results of the serological analyses, a Chi-square or Fisher’s exact test was conducted. Variables with a *p*-value ≤ 0.2 were selected for further analysis through robust Poisson regression in a multivariate model. To examine potential collinearity among the data, a correlation test was performed. If the correlation coefficient exceeded 0.9, one of the variables was removed based on biological plausibility criteria [21]. To evaluate the adequacy of the model, the Chi-square parameters and an Omnibus test were employed. The multivariate analysis was conducted at a significance level of 5% using SPSS version 23.0 software.

## 3. Results

Among the 387 animals analyzed, 23.5% (91/387; 95% CI: 18.8–27.2) tested positive for anti-*T. gondii* antibodies, with titrations ranging from 1:64 to 1:512. The seroprevalence of *N. caninum* was 1% (4/387; 95% CI: 0–2.7), with titrations ranging from 1:400 to 1:1600. Two animals (0.5%) tested positive for both infections (Table 1).

**Figure 1:**
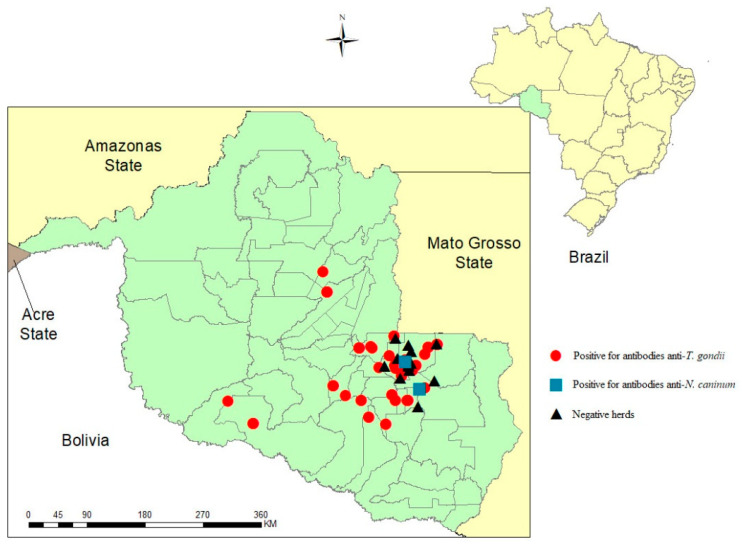
Geographical localization and serological status of anti-*T. gondii* and anti-*N. caninum* antibodies in 50 beef cattle herds from an Amazonian region of the State of Rondônia, North Brazil.

Among the 50 analyzed herds, 37 (74%) had at least one animal positive for anti-*T. gondii* antibodies. In two herds (4%), there were animals positive for anti-*N. caninum* antibodies, both of which were also positive for anti-*T. gondii* antibodies. The geographical locations and seropositivity status are shown in Figure 1.

From the univariate analysis, the significant variables (*p* ≤ 0.2) associated with *T. gondii* infections are presented in Table 2. However, no risk factors for animal infection were identified through multiple logistic regression. For *N. caninum* infections, no significant variables were found in the univariate analysis, indicating the absence of associated risk factors.

## 4. Discussion

The observed prevalence of 23.5% for anti-*T. gondii* antibodies was similar to that in studies conducted in South Brazil, where the prevalence rates were 29.1% in the State of Santa Catarina [22], 26% in the State of Paraná [23] and 17.4% in the State of Rio Grande do Sul [24]. In Southeast Brazil, specifically in the State of São Paulo, the prevalence was reported as 18% [25]. In Northeast Brazil, in the State of Paraíba, the prevalence was also 18% [26]. On the other hand, states closer to Rondônia showed higher prevalence percentages. For example, in Pará, located in North Brazil, the prevalence was 54.4% [18], while in Mato Grosso, in the Midwest region, it reached 71% [27]. In the eastern region of Rondônia, Souza et al. [14] reported a prevalence of 5.3%. It is important to note that the comparison of data can vary due to the use of different serological tests, variations in age, differences in sanitary management practices and regional factors that may contribute to the parasites’ life cycle, thereby increasing their transmission within herds.

The observed prevalence of anti-*N. caninum* antibodies was 1% (4/387). In Brazil, seropositivity values vary from 2.45% in the State of Mato Grosso [28] to 91.5% in the State of Minas Gerais [29]. In a previous study, the seroprevalence of *N. caninum* in the State of Rondônia was 10.4% [30], albeit using a lower cut-off of 1:25. This discrepancy in results could be attributed to differences in the diagnostic method protocols. It is important to note that lower cut-offs may result in an increased number of false-positive samples. Although there are debates about the ideal cut-off for the diagnosis of anti-*T. gondii* positivity in cattle, the most commonly used threshold is 1:200, which is considered more reliable [31,32,33,34].

The most frequent titers of anti-*T. gondii* antibodies were 1:64 (75.8%) and 1:128 (15.4%). Similar results were reported by Carmo et al. [35], who observed mostly low titers of 1:64 (55.2%) and 1:128 (33.5%). Cattle with low antibody titers may be in the chronic phase and could harbor viable cysts of the parasite in their tissues [36,37]. This phenomenon occurs because during acute infection, the immune response of the animal primarily involves CD4 Th1 cells targeting *T. gondii* tachyzoites. These tachyzoites then transform into bradyzoites and form cysts in a latent form to evade the immune system. Subsequently, a shift in the immune response occurs, leading to the predominance of CD4 Th2 cells, which aim to produce antibodies. As time progresses, antibody titers gradually decline to baseline levels since the body no longer requires the production of large quantities of antibodies [37,38].

Tissue cysts can remain viable for an indefinite period, representing the final stage in intermediate hosts, such as cattle [8]. It is important to note that meat from farmed animals is one of the primary sources of *T. gondii* infections in humans. However, the isolation of the parasite from cattle’s tissues is hindered by their strong resistance to infection. As beef is often consumed undercooked, it can pose a risk to the human population [8,39,40].

There was also a predominance of low titers of anti-*N. caninum* antibodies in the evaluated animals. In two animals, the titers were ≥1:800, which, according to Dubey [41], correspond to active infections, demonstrating that *N. caninum* is infecting cattle in the region, albeit in only 4% (2/50) of the evaluated herds.

The variable “contact with free-range chicken” showed significance in infection with *T. gondii* (*p* ≤ 0.05). Rizzo et al. [42] also reported an increased risk of *T. gondii* infection in sheep, associated with the presence of birds. Birds can attract hunting cats, which may subsequently excrete oocysts into the environment. According to Santos et al. [27], a single feline can shed and contaminate the environment with millions of oocysts. The cattle used in this study were raised under extensive management in a region surrounded by extensive forested areas. Therefore, the role of wild felines as potential transmitters of the parasite becomes noteworthy, particularly considering that 74% (37/50) of the assessed herds had at least one animal testing positive for anti-*T. gondii* antibodies.

Similarly, Chiebao et al. [43] conducted a survey to identify potential variables influencing the prevalence of *N. caninum*, and found that raising domestic poultry was a factor associated with infection in herds. Additionally, Rodrigues et al. [44] stated that domestic avians can serve as reliable indicators of the presence of *T. gondii* oocysts in the soil, making them valuable sentinel animals, particularly in areas with a high prevalence. This association can be attributed to the natural behavior of these animals, as they can mechanically transport oocysts from the environment to cattle’s food and water sources, thereby facilitating their dissemination.

The variable “pure breed” was selected in the univariate analysis of *T. gondii* infection (*p* ≤ 0.2). Biologically, there is limited understanding of the factors that could explain the correlation between purebred animals and *T. gondii* infections. Consistent with our own findings, Garcia et al. [45] observed a higher risk of toxoplasmosis infection among purebred Holstein cattle compared to crossbred animals. Similarly, Snak and Osaki [46] reported a significant association between purebred Jersey cows and seropositivity for anti-*N. caninum*, a pathogen similar to *T. gondii*. These observations highlight the potential influence of breed characteristics on susceptibility to these infections.

## 5. Conclusions

These study findings suggest a high prevalence of *T. gondii* infections among beef cattle in the Amazonian region of Rondônia, North Brazil. Considering the zoonotic nature of the parasite, the importance of cattle infections in the transmission of toxoplasmosis to humans should not be underestimated. However, the observed prevalence of *N. caninum* was relatively low. Conducting longitudinal studies would contribute to a better understanding of the factors influencing these prevalence values and aid in the development of more effective prevention and control measures.

## Figures and Tables

**Table 1:** Distribution of anti-*T. gondii* and anti-*N. caninum* antibody titration according to immunofluorescence antibody test (IFAT) in beef cattle intended for human consumption in an Amazonian region of North Brazil.

Positivity for Anti-*T. gondii* Antibodies				
Titration	1:64	1:128	1:256	1:512
Total (%)	69 (75.8)	14 (15.4)	6 (6.6)	2 (2.2)
**Positivity for Anti-*N. caninum* Antibodies**				
Titration	1:200	1:400	1:800	1:1600
Total (%)	-	2 (50)	1 (25)	1 (25)

**Table 2:** Univariate analysis of anti-*T. gondii* antibody positivity in beef cattle intended for human consumption in an Amazonian region of the North Brazil. Variables that present *p*-values ≤ 0.2 according to Chi-square or Fisher’s exact test.

Variable	Category	Total Animals	Positive (%)	*p*
Breed	Pure	205	59 (28.8)	0.149
	Mixed	182	32 (17.6)	
Contact with free-range chicken	No	201	39 (19.4)	0.049
	Yes	186	52 (28)	

## Data Availability

Not applicable.
